# Reperfusion Therapies and Post-Stroke Seizures: Evidence, Mechanisms and Clinical Implications—A Narrative Review

**DOI:** 10.21315/mjms-03-2025-166

**Published:** 2025-08-30

**Authors:** Erum Shariff

**Affiliations:** Department of Neurology, College of Medicine, Imam Abdulrahman Bin Faisal University, Dammam, Saudi Arabia

**Keywords:** ischaemic stroke, reperfusion therapies, post-stroke epilepsy, post-stroke seizures, haemorrhagic transformation, acute symptomatic seizures, late seizures, pathophysiological mechanisms

## Abstract

Seizures are a frequent complication of stroke and are often linked to worse functional outcomes. With advancements in acute stroke care, particularly the use of reperfusion therapies (RTs) such as intravenous thrombolysis, intra-arterial therapies and mechanical thrombectomy, the relationship between these treatments and post-stroke seizures (PSS) requires re-evaluation. This review explores the mechanisms connecting RTs to acute symptomatic seizures (ASS) and post-stroke epilepsy (PSE), summarises current evidence on their association and identifies gaps in knowledge to guide future research. A literature search was conducted using PubMed, Scopus, Cochrane and Google Scholar, focusing on terms such as “seizures,” “epilepsy,” “stroke,” “thrombolysis,” “rtPA,” “reperfusion therapies,” and “thrombectomy.” The reported frequency of seizures following thrombolysis was 4%–15%, with RTs potentially influencing PSS through complex, sometimes opposing, mechanisms. While some studies suggested that RTs could increase seizure risk due to factors like reperfusion injury or haemorrhagic transformation, others proposed a protective effect by reducing infarct size and stroke severity. Current evidence does not confirm a strong link between RTs and PSS, and most research has focused on ASS rather than PSE. The pathophysiology of seizures in this context remains unclear, with multiple contributing factors. The effect of RTs on PSS is poorly understood, highlighting the need for better definitions of seizures, standardised monitoring and high-quality, multicentre prospective studies. Leveraging stroke registries and addressing clinical challenges, such as stroke characteristics and reperfusion success, are critical to clarifying this relationship and improving stroke care.

## Introduction

Cerebrovascular accidents represent one of the foremost contributors to worldwide disability and fatality rates, disproportionately affecting resource-limited countries. These countries account for approximately nine-tenths of both stroke-related disability burdens and mortality cases globally (1–3). Cerebrovascular events and their epileptic complications are a major cause of acquired neurological morbidity, especially in ageing populations. The development of seizures following stroke is correlated with diminished functional rehabilitation, reduced quality of life indicators and increased mortality rates, highlighting their clinical significance as negative prognostic markers (3).

Therefore, identifying the predictors of post-stroke seizures (PSS) and developing strategies for their early detection and prevention are of critical clinical importance. Recent advances in stroke management, particularly reperfusion therapies (RTs), such as intravenous recombinant tissue plasminogen activator (rtPA) and mechanical thrombectomy (MT), have revolutionised acute ischaemic stroke (IS) treatment, leading to a 68% increase in stroke survival rates (4–6). However, as more patients survive strokes, the incidence of PSS has emerged as a growing concern, necessitating further research and clinical attention (7).

Although intravenous thrombolysis (IVT) enhances functional recovery and reduces mortality in stroke patients, emerging evidence suggests its association with both acute symptomatic seizures (ASS) and increased epileptiform discharges on electroencephalogram (EEG) monitoring (8–10). Notably, seizures have been reported during rtPA administration, even in the absence of visible cerebral lesions, as seen in cases of thrombolysis for acute myocardial infarction (10). This raises the possibility of a direct link between thrombolysis and seizures, which, if confirmed, could have significant clinical implications.

In addition, rtPA carries inherent risks, including a 4%–6.4% risk of haemorrhagic transformation (HT), which can lead to fatal intracranial haemorrhage in approximately 40% of affected patients (11, 12). These risks underscore the need for careful patient selection and monitoring during rtPA administration. For large vessel occlusions (LVO), endovascular intervention has distinct advantages over systemic thrombolysis, with significantly improved reperfusion success and enhanced clinical recovery metrics (13–16). As a result, MT has become the preferred treatment for acute IS in many cases, particularly when rtPA is contraindicated or insufficient. However, the relationship between MT and PSS remains poorly understood, warranting further investigation.

Seizures manifesting within seven days post-stroke are categorised as ASS, whereas events occurring beyond this period are designated as either late seizures or post-stroke epilepsy (PSE) (17, 18). Current diagnostic criteria recognise that even a single late seizure episode may meet the threshold for PSE diagnosis under established international guidelines (19).

Despite these classifications, the mechanisms linking RTs to ASS and PSE remain unclear, highlighting a critical gap in the literature. Thus, this narrative review investigates two key propositions: i) whether RTs demonstrate an independent association with PSS and ii) the potential biological mechanisms connecting these interventions to both acute and delayed post-stroke epileptogenesis.

By analysing existing literature, the researcher aims to elucidate the association between RTs and PSS and emphasise its relevance to neurologists, epileptologists and stroke care specialists. Our goal is to identify gaps in understanding and stimulate further research to inform more effective management strategies for PSS, ultimately improving outcomes for stroke survivors.

## Methods

To ensure a rigorous evidence base, researcher systematically searched five major biomedical databases (PubMed, Scopus, Cochrane Library, Google Scholar and Web of Science) using predefined search strategies. This multidatabase approach facilitated the identification of relevant publications across the spectrum of stroke and epilepsy research. The search aimed to gather the most current and relevant evidence on the association between acute stroke RTs and PSS, with the final search conducted on 10 October 2024. The review also included a cross-reference search and a review of the personal library to ensure a thorough capture of the studies.

The database queries used Boolean operators to combine key terms representing: i) seizure disorders (“seizures”/”epilepsy”); ii) cerebrovascular pathology (“stroke”/”cerebrovascular disease”) and (“thrombolysis”/”rtPA”/”reperfusion therapies”/”thrombectomy”); and iii) intervention modalities. To focus on recent and relevant literature, the search was restricted to articles published between 2013 and 2024. This approach ensured the inclusion of studies that are current and that provide a comprehensive understanding of the topic.

The researcher critically appraised all included studies by extracting essential methodological parameters, including study design, cohort size and analytical approach. The Newcastle–Ottawa Scale (20) was systematically applied to evaluate potential biases and overall study quality. As this was a narrative review based solely on existing literature, ethics committee approval was not required. The review relied on publicly available studies and did not involve direct patient data or new experimental research.

## Results

In this narrative review, ASS and PSE are discussed collectively as PSS. Table 1 summarises data from a diverse range of studies conducted across multiple countries, including China, India, Austria, Sweden, Germany, the United States, Qatar, Italy, Portugal, Switzerland, Spain, France, Brazil, Israel, Australia, Japan and the Netherlands. These studies employ various methodologies, such as retrospective, cohort and prospective designs, offering a comprehensive global perspective on the relationship between RTs and PSS. The mean or median age of patients across studies varies significantly, ranging from 40 to over 76 years, reflecting regional and population differences. A consistent male predominance is observed in both stroke incidence and treatment outcomes, although the percentage of male participants differs between studies. Sample sizes also vary widely, from 28 to 595,545 participants, highlighting the diversity in research scales and contexts.

The reported frequency of seizures following thrombolysis ranges from 4% to 15%, consistent with rates observed in the pre-thrombolytic era (21). The incidence of ASS after RTs shows considerable variability, with the lowest reported incidence being 0.3% in low-dose rtPA (21). These findings underscore the complexity of the relationship between RTs and PSS, emphasising the need for further research to clarify this association.

### Potential Pathophysiological Mechanisms Linking Thrombolysis to Seizures

Thrombolysis has been linked to seizures, with studies suggesting that it may have proconvulsive and epileptogenic effects. Restoring blood flow post-thrombolysis could increase cortical excitability, potentially triggering seizures (8, 9, 44, 45, 46). Experimental and clinical evidence highlights the neurotoxic and epileptogenic role of rtPA, which may alter brain excitability and increase seizure risk after IS. Animal studies have shown that mice lacking endogenous rtPA are less prone to seizures, while those overexpressing rtPA have a lower seizure threshold (44, 46). This is thought to occur through rtPA’s upregulation of N-Methyl-D-Aspartate (NMDA) receptors, which lowers the brain’s hyperexcitability threshold and creates a seizure-prone environment. Thus, rtPA, although critical for stroke treatment, may also act as a proconvulsive agent in some individuals (45).

### Proconvulsant and Neurotoxic Effects of RtPA

#### rtPA-induced Loss of Gamma-aminobutyric Acid (GABA)ergic Interneurons

The loss of inhibitory interneurons, especially those mediated by GABA, is linked to seizure development. GABA is essential for balancing excitatory and inhibitory signals in the brain, and its dysfunction can lead to neuronal hyperexcitability (47).

### Upregulation of Matrix Metalloproteinases (MMPs) by rtPA

MMPs, particularly MMP-9, play a key role in stroke pathophysiology and PSS. rtPA elevates MMP-3 and MMP-9 activity via direct proteolytic activation. MMP-3 induces vascular instability through the degradation of extracellular matrix components, while MMP-9 contributes to neurovascular dysfunction by increasing blood brain barrier (BBB) permeability and facilitating neurotoxin extravasation. These effects worsen neuronal injury, inflammation and seizure risk following IS and thrombolytic therapy (48).

### Anticonvulsant and Neuroprotective Effects of rtPA

Despite its potential to disrupt the BBB and contribute to neuronal injury, rtPA also demonstrates neuroprotective effects under certain conditions. These benefits are mediated through the following mechanisms that help mitigate ischaemic damage and promote stroke recovery:

- Activation of brain-derived neurotrophic factor (BDNF): rtPA promotes the activation of BDNF, which supports neuronal survival, plasticity and repair. By enhancing BDNF signalling, rtPA may reduce cell death, protect against ischaemic injury and improve recovery. This stabilisation of the neural environment could also lower the risk of seizures, counteracting some of the harmful effects of stroke and RTs (49).
- Inhibition of apoptosis: At low concentrations, rtPA inhibits apoptotic cell death, preserving neuronal integrity and function after IS. These lower doses are also linked to anticonvulsive properties, potentially reducing seizure risk. This dual role is neuroprotective at low doses and proconvulsive at higher doses, underscoring rtPA’s complex, dose-dependent effects on brain function post-stroke (50).

### Investigating the Connection between Thrombolysis and the Occurrence of Seizures

Several clinical studies have indicated a potential connection between thrombolysis and seizures (6, 8–10, 51–53). One proposed mechanism is reperfusion/hyperperfusion syndrome. Hafeez et al. (10) described seizure activity during rtPA infusion in two patients, which was potentially mediated by abrupt cortical reperfusion following arterial recanalisation. This phenomenon mirrors the seizure pathogenesis observed in hyperperfusion syndromes, in which sudden cerebral blood flow restoration triggers neuronal hyperexcitability commonly observed after carotid endarterectomy (54).

The proposed “stunned brain” phenomenon (55) represents another plausible contributor to elevated seizure susceptibility following cerebral ischaemia–reperfusion injury. This syndrome describes a temporary, reversible state in which the brain remains impaired after an ischaemic event despite early vessel recanalisation. Clinical improvement is often delayed, with recovery taking months. Notably, nearly 40% of patients who showed no immediate response to revascularisation still achieved favourable outcomes by three months, suggesting the presence of this delayed recovery phenomenon (55). It parallels the “stunned myocardium,” in which prolonged dysfunction occurs after reperfusion following coronary ischaemia (56).

The link between thrombolysis and seizures may also stem from the unique imaging features in rtPA-treated patients. Secondary cortical infarction due to distal embolisation or islands of viable cortical tissue within the infarct zone are known risk factors for PSS (57). In addition, the increased risk of HT, a common complication of rtPA therapy, may further elevate seizure likelihood. HT worsens brain injury, disrupts neuronal function and increases tissue excitability, thereby increasing the risk of PSS (58).

Moreover, the higher reported incidence of seizures in patients undergoing RTs may be influenced by more intensive clinical monitoring in the acute post-stroke phase, lowering the detection threshold for seizures. However, RTs may also reduce the risk of PSS by limiting stroke severity, which is a major risk factor for PSS (58). By minimising brain injury, RTs could play a protective role against epileptogenesis and the development of PSE. For example, Chen et al.’s (59) retrospective analysis demonstrated that early IVT administration was associated with a decreased incidence of PSE. Thus, while thrombolysis may increase seizure detection in the short term, it may also lower long-term seizure risk by reducing stroke severity and brain injury.

### RTs and Various Types of PSS: Investigating the Clinical Association

The subsequent sections will examine the clinical evidence of the potential link between RTs and the occurrence of both ASS and PSE. Researcher will explore possible pathophysiological mechanisms, compare different reperfusion strategies and discuss their implications for clinical practice.

### Studies with a Main Focus on ASS

The relationship between RTs and ASS varies, underscoring the individualised nature of these therapies’ effects on seizure development. Xu et al. (21) reported differential ASS rates between thrombolytic regimens, with standard-dose IVT demonstrating a 1.1% incidence versus 0.3% for low-dose protocols. This difference highlights the importance of considering factors such as rtPA dosage and patient-specific characteristics when assessing post-reperfusion seizure risk. Brigo et al.’s (23) case-control investigation established cortical involvement (adjusted odds ratio [OR] = 2.49) and IVT administration (adjusted OR = 2.26) as significant independent predictors of ASS. In a follow-up study, Brigo et al. (22) found that ASS in RT-treated patients were predominantly focal aware or generalised convulsive seizures. The authors highlighted a critical knowledge gap—whether the observed low incidence of focal impaired awareness seizures and nonconvulsive seizures/status epilepticus reflects true epidemiological rarity or underdiagnosis due to detection limitations in acute post-stroke settings. Further studies are needed to clarify this diagnostic uncertainty.

Interestingly, more than 20% of patients with PSS exhibit only electrographic seizures, underscoring the importance of further exploration into nonconvulsive seizures and improving their detection during the acute post-stroke period (60). Tako et al. (24) found no difference in PSS rates between IVT, MT and combined treatments. However, a high National Institutes of Health Stroke Scale (NIHSS) score 24 h post-admission was a key predictor of ASS, emphasising the role of early stroke severity in seizure risk. Kohlhase et al.’s (25) retrospective cohort study (*N* = 987) demonstrated no significant association between LVO revascularisation and subsequent ASS development using MT, IVT or a combination of both.

Mushannen et al. (26) conducted a comparative analysis of 508 patients receiving RTs versus non-treated controls and demonstrated that MT, IVT or their combined intervention did not elevate ASS risk in IS patients. Ba et al. (27) studied 1,638 patients to assess how rtPA is linked to MT and ASS. Using propensity score matching to reduce confounding, they found no significant association between rtPA and ASS incidence. Agarwal et al. (28) conducted a prospective cohort study and found no significant link between IVT or MT and ASS, suggesting that rtPA’s neurotoxic effects could be offset by its ability to reduce infarct size, a key factor in ASS development.

Agarwal et al.’s (28) prospective cohort study identified no significant association between IVT or MT and ASS. The authors proposed that rtPA’s potential neurotoxic properties could be counterbalanced by its neuroprotective effect through infarct volume reduction, a critical determinant of seizure risk. Agashe et al. (29) retrospectively analysed 98 patients undergoing MT in an extended time window (6 h to 24 h) and found a 4.1% incidence of ASS. The analysis revealed comparable ASS rates between treatment groups, but patients experiencing ASS demonstrated significantly worse functional recovery at a 90-day follow-up compared with their seizure-free counterparts.

Zöllner et al. (30) conducted a retrospective case-control study of 135,117 patients, including 13,356 patients treated with IVT alone and 1,013 patients treated with both IVT and MT. The study found no significant increase in ASS in patients receiving IVT or combined IVT/MT compared with the controls. Belcastro et al. (7) conducted a prospective study of 516 patients and found that 3.8% of those receiving RTs developed ASS, compared with 2.3% in the non-RT group (*P* = 0.45). They observed no significant difference in ASS incidence between the two groups and found comparable rates of ASS across both treatment cohorts.

Anadani et al. (31) studied 459 patients undergoing MT, with 92.8% achieving successful recanalisation (TICI ≥ 2b). Only 2.4% (11 patients) developed ASS. The regression model demonstrated that the Alberta Stroke Programme Early Score of ≤ 6 independently predicted seizure occurrence following a stroke. In addition, ASS were significantly associated with increased 90-day mortality rates and worse functional recovery outcomes. Alvarez et al.’s (8) retrospective analysis (*N* = 2,327) reported a 1.2% incidence of ASS and identified thrombolytic administration and cortical lesion location as significant predictors following RTs. By contrast, Jiang et al. (61) proposed that MT could reduce acute seizure risk without altering the overall epilepsy incidence.

### Studies with a Main Focus on PSE

The observed 2%–14% cumulative risk of developing epilepsy after stroke necessitates individualised prevention approaches based on validated risk factors (62). Eriksson et al.’s (32) population-based study (*N* = 3,319) found significant variability in PSE development based on treatment modality. MT was associated with a substantially reduced risk (hazard ratio [HR] = 0.51) compared with the untreated controls. The overall cohort exhibited a 7.9% PSE incidence, with stratified analysis revealing 10.0% (95% confidence interval [CI] = 8.3–11.8) for IVT monotherapy, 6.5% (95% CI = 5.3) for IAT and 12.3% (95% CI = 10.3–14.3) for untreated patients.

Survival-adjusted analyses showed that untreated patients had nearly twice the two-year PSE risk of IAT-treated cases. Unsuccessful recanalisation, HT, large infarctions and hemicraniectomy increased PSE risk, whereas IVT before IAT and no infarction on 24-h computed tomography (CT) reduced it. IAT was linked to a lower PSE risk than IVT or no treatment, lasting up to four years, regardless of age or stroke severity (32).

Nesselroth et al.’s (33) retrospective analysis (*N* = 234) demonstrated a 66.4% relative reduction in seizure incidence with rtPA compared with antiplatelet therapy. Although constrained by methodological limitations, these findings align with emerging evidence suggesting a potential neuroprotective effect of thrombolysis against PSE (34, 63). Gruber et al.’s multicentre data (35) showed: i) a 9% cumulative PSE incidence post-thrombectomy and ii) no significant risk modification with combined IVT, consistent with prior studies (32, 36, 53). Larger infarct sizes and cerebral microbleeds on post-interventional magnetic resonance imaging were identified as independent PSE risk factors.

Thevathasan et al. (37) analysed 205 patients treated with IVT and IAT and found that those with HT after IAT were nearly five times more likely to develop PSS within two years. This study suggests that HT could be an imaging biomarker for predicting PSS. Kuohn et al. (20) observed a significantly elevated PSE risk across all 14 reperfusion strategies, with both IVT and MT showing clinically important hazard ratios. The observed effect persisted in the combined treatment cohorts.

Keller et al.’s (34) investigation revealed a 13.9% incidence of PSE following IVT, underscoring the long-term neurological risks associated with RTs. Multivariable regression analysis identified five independent predictors of epilepsy development: impaired functional status at discharge (low Barthel Index score), presence of visual field deficits, nosocomial infections during hospitalisation, temporal lobe involvement and perirolandic cortical involvement. While unadjusted analysis showed greater PSE frequency in IVT-treated patients (20.6% vs. 10.7%, *P* = 0.020), a multivariate adjustment for treatment covariates attenuated this association to non-significance. The final model showed that IVT was not an independent predictor of PSE development.

### Studies Primarily Focused on Both ASS and PSE

Bentes et al.’s (6) prospective comparative study evaluated epileptic events, clinical seizures and EEG abnormalities in IS patients with and without IVT. The investigation revealed comparable rates of ASS, PSE and epileptiform EEG patterns between treatment cohorts. These findings suggest that thrombolytic therapy does not modify seizure risk, potentially due to balanced baseline characteristics, including comparable stroke severity and cortical involvement distribution following treatment allocation.

Naylor et al.’s (38) seven-year retrospective analysis of anterior circulation IS patients compared seizure incidence across four treatment cohorts: IVT alone, IAT alone, combined therapy and conventional stroke unit management. After adjusting for age, stroke severity and functional outcomes, multivariable regression demonstrated significantly increased seizure likelihood across all reperfusion strategies relative to standard care alone. Notably, IAT alone showed a threefold increase in seizure risk, independent of age, stroke severity or functional outcomes. While rtPA has been linked to neurotoxicity, the higher seizure risk with IAT suggests that reperfusion, rather than rtPA, may be the key factor. These findings highlight a potential increased risk of PSS with RTs, particularly IAT, underscoring the need for seizure monitoring post-reperfusion.

However, the study’s retrospective design and limitations, such as the closer monitoring of RT patients (potentially increasing seizure detection), the lack of prospective EEG recordings and the absence of separate data on ASS and PSE, may explain the discrepancies with Bentes et al.’s findings. In addition, the study did not account for other seizure risk factors, such as hypertension, lesion size or cortical involvement.

Ishihara et al.’s (39) recent retrospective analysis of 237 LVO cases revealed an ASS rate of 5.1% and a PSE incidence of 4.2% following IVT or MT. The study found no statistically significant elevation in seizure risk attributable to RTs. Ferreira-Atuesta et al.’s (40) multicentre investigation applied propensity score matching in 4,229 participants to evaluate seizure risk following RTs. The matched analysis found no significant association between treatment exposure and either ASS or PSE development.

Alemany et al. (36) conducted a retrospective study on IS patients with an NIHSS > 8 who underwent MT. The study included 344 patients, of whom 6.1% developed ASS and 4.12% experienced PSE within the first year. By five years, the incidence decreased to 1.6%, with a cumulative incidence of 8.93%. They found that ASS was associated with PSE but that MT alone did not increase the risk of either ASS or PSE. Alemany et al. (36) retrospectively analysed 344 moderate-to-severe IS patients (NIHSS > 8) treated with MT and reported a 6.1% incidence of ASS and a 4.12% PSE rate within the first year. The annual seizure incidence declined to 1.6% by five years, yielding a cumulative epilepsy risk of 8.93%. Although ASS were associated with subsequent epilepsy development, the study found no evidence that thrombectomy independently increased the risk of either acute or late-onset seizures.

Eriksson et al. (41) evaluated 90 thrombectomy-treated patients and identified a 4.4% ASS rate and a 5.3% two-year PSE risk, with no significant association between MT and either outcome. By contrast, Brondani et al.’s (42) IVT cohort (*N* = 153) demonstrated a higher seizure incidence, with 13.7% experiencing acute events and 9.8% developing epilepsy.

### RTs, PSS and Their Impact on Functional Outcomes

Table 2 summarises the findings from studies on the effect of RTs on ASS, PSE and outcomes related to mortality and the modified Rankin Score (mRS) in IS patients. Mortality and mRS outcomes varied. For example, Xu et al. (21) found higher, although not statistically significant, in-hospital mortality with low-dose IV-rtPA. Anadani et al. (31) identified ASS as a significant prognostic marker and demonstrated their association with increased 90-day mortality rates and worse functional recovery. Naylor et al. (38) noted significant differences in mRS scores across therapy groups. Lei et al. (64) found that patients with an ASPECT score ≤ 5 could still achieve favourable outcomes after MT, although those with complications, such as intracranial haemorrhage or high NIHSS scores, had poorer outcomes.

Agashe et al. (29) demonstrated that patients experiencing seizures following MT had an eight-fold increased likelihood of unfavourable functional outcomes (mRS > 2) at a 90-day follow-up. Brondani et al. (42) found that 14.3% of IV-rtPA patients died. While, Bentes et al. (6) observed higher 12-month mRS scores in IV-rtPA patients. Kohlhase et al. (25) suggested that MT and IVT reduced mortality. These findings emphasise the importance of personalised treatment approaches to optimise outcomes based on individual patient profiles and responses.

## Discussion

This review explores the relationship between RTs for IS and PSS, specifically ASS and PSE. Our analysis of the existing clinical evidence reveals a lack of a consistent and solid link between RTs and PSS. Several studies have reported varying associations, similar to the conclusions from previous systematic reviews (53, 61, 66). A key methodological concern in studies examining PSS is confounding by treatment selection. Patients with severe strokes, particularly those involving LVO, are more likely to receive RTs and simultaneously face an elevated risk of seizures due to the extent of cortical injury (7, 22, 30, 38). This complicates causal inferences regarding treatment effects. Another issue is the lack of radiological biomarkers. Despite advances in neuroimaging, no validated biomarkers currently exist to predict epileptogenesis (i.e., the progression to chronic epilepsy) after stroke (67). However, structural imaging findings can provide prognostic clues: Cortical involvement is strongly associated with subsequent epilepsy, while purely subcortical strokes do not significantly elevate the risk (68).

The vascular territory of the stroke also plays a critical role. Anterior circulation strokes, particularly those affecting the middle cerebral artery territory, substantially increase seizure susceptibility (69, 70), while infratentorial strokes are seldom implicated. Infarct volume serves as a critical risk modifier, with large ischaemic lesions (≥ 70 mL volume) demonstrating a fourfold increased probability of PSS (71). While cerebral microbleeds initially showed an association with ASS, the multivariate adjustment for stroke severity, cortical localisation and haemorrhagic complications attenuated this relationship, indicating that these covariates could mediate the observed correlation (72). Such validated parameters form the foundation of the SeLECT prediction model, which exhibits strong discriminative performance in forecasting epilepsy risk following IS (73). Therapeutic interventions for secondary stroke prevention may introduce significant bias in PSE risk estimation. Antithrombotic agents, antihypertensives and neuroprotective medications could potentially modify seizure risk through either direct pharmacological effects or by altering the underlying cerebrovascular pathophysiology.

The potential for confounding by treatment indication remains a significant methodological concern, as therapeutic decisions are inherently guided by stroke characteristics (severity, underlying aetiology and comorbid conditions) that independently influence epileptogenesis. This interdependence complicates the isolation of treatment effects from baseline risk factors in PSE studies. While observational studies have suggested a potential reduction in ASS among statin users (74, 75), these findings require cautious interpretation due to significant methodological limitations. The observed associations may be confounded by indication, as statin prescription patterns often reflect underlying cardiovascular risk profiles rather than direct PSS prevention or antiepileptic effects. Furthermore, treatment selection bias may occur if statins are preferentially prescribed to patients with specific stroke characteristics or severity levels. These inherent limitations of observational designs underscore the need for prospective controlled trials to establish causal relationships and elucidate potential neuroprotective mechanisms of statins.

While the impact of RTs on the development of PSS remains inconclusive, it is evident that these therapies, including IVT and MT, significantly improve survival and functional outcomes. However, their potential role in inducing or modulating the risk of seizures is complex and potentially opposing. Stroke is a significant global health concern, with IS posing a notably higher risk for seizures compared with haemorrhagic strokes (76). RTs, including IVT and endovascular therapies, such as IAT and MT, remain the gold standard in acute IS treatment. These RTs offer substantial improvements in long-term survival and functional recovery but also carry risks, including the induction of PSS (77). This review aims to bridge the gap in the literature by examining the relationship between acute stroke RTs and the development of ASS and PSE.

### Comparing Reperfusion Strategies

Current evidence shows considerable variability regarding seizure risks across RTs. While multiple investigations, including Belcastro et al. (7) and Kohlhase et al. (25), have reported comparable seizure rates between treatment strategies, Ferreira-Atuesta et al. (40) identified elevated ASS odds with both IVT and MT. Conversely, Bentes et al. (6), Anadani et al. (31) and Brondani et al. (42), documented numerically reduced, although not statistically significant, seizure frequencies following revascularisation therapies. A review by Lekoubou (66) suggested that around 1 in 15 IS patients develop seizures regardless of RTs. Furthermore, data revealed that the incidence of PSS was 6.1% following IVT, 5.9% after MT and 5.8% with combination therapy, indicating that certain RTs may independently increase the risk of PSS. These findings raise important questions about potential treatment-specific epileptogenic effects, although the underlying pathophysiological mechanisms require clarification, and future investigations should address critical knowledge gaps. Eriksson et al. (32) and Gruber et al. (35) observed divergent PSE rates depending on treatment modality, while Kuohn et al. (20) identified elevated risk to be specifically associated with IV-tPA and MT. Ferreira-Atuesta et al. (40) reported increased PSE likelihood following IVT compared with untreated controls. This contrasts with findings from Keller et al. (34), Brondani et al. (42), Arntz et al. (43) and Chen et al. (65), whose studies revealed widely varying PSE rates after IVT. Notably, Bentes et al. (77) found no robust association between RTs and PSE development, with only limited evidence supporting such a relationship. Collectively, these studies underscore the complexity of the RT–PSE association, which appears modulated by multiple factors, including treatment selection, follow-up duration and baseline patient characteristics.

### Strengths and Limitations of the Review

The strength of this review lies in its thorough examination of the global relationship between acute stroke RTs and PSE. However, several limitations must be acknowledged, including variability in study designs and the potential for biases and inconsistencies in defining and measuring outcomes, such as early seizures and PSE. These factors make it challenging to draw definitive conclusions and highlight the need for more standardised protocols in post-stroke care and research.

### Challenges in Study Design and Methodology

It is crucial to recognise that the studies investigating RTs and PSS often used retrospective and case-control designs (8, 20, 22, 32, 33, 35, 37, 38). These designs are prone to biases, such as selection bias, recall bias and confounding factors, which may affect outcomes. Retrospective studies depend on pre-existing data that may be incomplete or inaccurate, while case-control studies may suffer from unrepresentative control groups, especially in stroke research, in which thrombolysis-treated patients may differ significantly from the broader stroke population. The relatively low incidence of ASS necessitates large sample sizes to accurately estimate their frequency and to investigate the link between RTs and seizures.

In this context, case-control studies may offer greater sensitivity for detecting rare events, but the literature on treatments such as IAT, thrombolysis and MT remains limited, making definitive conclusions difficult. Variability in procedural techniques, stroke management practices and patient care across medical centres further complicates the issue. Therefore, large-scale meta-analyses that aggregate data from multiple studies are essential for clarifying any potential connections between RTs and PSS.

### Diagnostic Challenges and EEG Monitoring

The diagnosis of PSS is challenging, particularly in distinguishing between epileptic and other neurological events (68, 78, 79). Misdiagnosis is a risk due to the overlap of seizure-like symptoms with other conditions, complicating the diagnosis. In addition, the limited use of EEG in routine clinical practice may lead to the under-detection of subclinical or nonconvulsive seizures. Evidence suggests that nonconvulsive status epilepticus affects approximately 3.8% of IS patients, although this figure may be underestimated due to underdiagnosis without EEG assessment (60, 70, 71, 80). Given the challenges in clinical detection; particularly for subtle or nonconvulsive seizures, future prospective studies incorporating systematic EEG monitoring are essential to assess seizure frequency and improve our understanding of PSE.

### Future Research Directions

To address the challenges in investigating the impact of RTs on PSS, future research must control for various factors influencing seizure occurrence, including stroke severity, haemorrhagic complications and the involvement of cortical regions (81). Standardising clinical and neurophysiological monitoring across patient groups, including those treated with RTs and those who are not, can help mitigate performance biases. To elucidate the complex relationship between stroke interventions and seizure outcomes, rigorously designed, multicentre prospective studies are imperative.

Such investigations should incorporate an extended longitudinal follow-up (> 2 years) to capture delayed epileptogenesis, standardised EEG monitoring protocols for seizure detection, large and diverse cohorts powered for subgroup analyses and the integration of multinational stroke registry data for enhanced generalisability. This approach will enable a more precise characterisation of treatment-associated risks while controlling for confounding by stroke severity, localisation and comorbidities, ultimately informing personalised therapeutic decision-making.

## Conclusion

In conclusion, the question of whether RTs independently linked to PSS remains unresolved. Inconsistent results and methodological limitations necessitate higher-quality studies to determine if RTs independently increase seizure risk and through what biological pathways. To advance our understanding, PSS should be thoroughly investigated through well-designed, high-quality, and multicentric prospective studies within the evolving landscape of stroke care.

## Figures and Tables

**Table 1:** Baseline characteristics of included studies

Author and year of publication	Study design	Number of controls	Number treated with RTs	Type of RTs	Type of -PSS	Association between RTs and PSS	Age in years (Mean ± SD) or Median (IQR or Range)
Bentes et al. 2017 (6)	Prospective study	151	107	IV-rtPA	ASS	No	69.64 ± 10.55
			44	Control	PSE		62.72 ± 13.26

Belcastro et al. 2020 (7)	Observational prospective study	516	153	IVT	ASS	No	71 (20–96)
			58	IV-rtPA + MT			
			81	MT			
			254	No RT			

Alvarez et al. 2013 (8)	Case-control study	2,327	2,327	IVT	ASS	Yes	70.9, SD = NA

Kuohn et al. 2022 (20)	Retrospective study	595,545	22,818	IV-rtPA	PSE	Yes	74 (62–83)
			802	MT	PSE	Yes	
			1,139	IV-tPA + MT	PSE	Yes	
			570,786	No RT			

Xu J et al. 2023 (21)	Retrospective comparative study	1,334	368	Low-dose IVT	ASS	No	67.2 ± 11.6

Brigo et al. 2020 (22)	Retrospective case-control		558	IVT	ASS	Yes	71 (20–96)
			128	IAT ± MT	ASS	No	

Brigo et al. 2020 (23)	Retrospective case-control			IVT	ASS If IVT with cortical involvement	Yes	72.94 ± 9

Tako et al. 2022 (24)	Retrospective study	979		Overall	ASS	No	72.3 ± 11.6
			430	IV-rtPA			
			472	MT			
			264	IV-rtPA + MT			
			403	MT with TICI ≥ 2b			

Kohlhase et al. 2022 (25)	Propensity scores matching study Retrospective analysis	987	208	MT+/IV-rtPA	ASS	No	70 ± 14
			264	MT+/IV- rtPA+			69 ± 14
			169	MT /IV- rtPA+			75 ± 13
			346	MT /IV- rtPA			76 ± 12

Mushannen et al. 2021 (26)	Retrospective matched case-control study		342	IV-tPA	ASS	No	53.3 ± 13.1
			70	MT			
			96	IV-tPA + MT			
			501	Control			

Ba et al. 2021 (27)	Prospective observational study	1,638	1,007	-rtPA	ASS	No	71 (60–82)
			3	MT+IV-rtPA			
			8	MT+IV-rtPA			

Agarwal et al. 2021 (28)	Prospective cohort study	291	22	rtPA	ASS	No	51.5 ± 14.5
			7	MT			

Agashe et al. 2021 (29)	Retrospective study (extended window)		98	MT	ASS	No	NA

Zöllner et al. 2020 (30)	Retrospective case-control study		14,323	IVT	ASS	No	75.8 ± 11.6
			1,060	IVT+MT+			
			536	MT			

Anadani et al. 2019 (31)	Retrospective study		459	MT	ASS	Yes	67.5 ± 15.1
					If ASPECT < 6		

Eriksson et al. 2023 (32)	Case-control study		3,319	MT	PSE	No	73–74
			3,132	IVT			
			3,184	Control			

Nesselroth et al. 2018 (33)	Non-randomised retrospective case-controlled study		234	IV-rtPA Control	LS	No	68.24, SD = NA

Keller et al. 2015 (34)	Retrospective study		302	IV-rtPA	PSE	No	68 (57–76)

Gruber et al. 2023 (35)	Retrospective cohort study		348	MT	PSE	Yes	67 (18–89)

Alemany et al. 2021 (36)	Retrospective study			MT	ASS PSE	No	66.5 ± 11.5

Thevathasan et al. 2018 (37)	Retrospective (case-control)	149 without HT	56 with HT	IAT with or without IVT	ASS PSE	Yes	69 (57–78)

Naylor et al. 2018 (38)	Retrospective, multicentre cohort study	1,375	112	IAT + IV-rtPA	ASS PSE	Yes	70 (59–78)
			93	IAT			67 (54–77)
			363	IV-tPA			74 (65–82)
				Control			62 (52–70)

Ishihara et al. 2024 (39)	Retrospective comparative study	237	74	IV-tPA + MT+	ASS	No	79–83 (82)
			82	MT only			
			28	IV-tPA only			
			53	IV-tPA – MT-			

Ferreira-Atuesta et al. 2021 (40)	Matched study	4,229	1,225	RT (IVT, IAT, MT)	ASS PSE	No	72 (61–80)

Eriksson et al. 2020 (41)	Retropsective		90	MT	ASS PSE	No	72 (65–80)

Brondani et al. 2019 (42)	Retrospective cohort study		153	IV-rtPA	ASS PSE	Yes	67.24 ± 13.13

Arntz et al. 2013 (43)	Prospective cohort study	537		IV-rtPA	PSE	No	40.0 ± 8.0

RT = reperfusion therapies; PSS = post-stroke seizure; IV = intravenous; tPA = tissue plasminogen activator; MT = mechanical thrombectomy; IVT = intravenous thrombolysis; IAT = intra-arterial thrombolysis; rtPA = recombinant tissue plasminogen activator; TICI = thrombolysis in cerebral infarction; ASS = acute symptomatic seizure; PSE = post-stroke epilepsy; ASPECT= Alberta Stroke Programme Early Score; LS = late seizures; NA = not applicable

**Table 2:** Incidence of ASS, PSE and adverse outcomes following ischaemic stroke RTs

Author and year of publication	Type of RTs	Sample size	Follow-up time	ASS *n* (%)	*P*-value/OR (95% CI)/ HR (95% CI)	PSE	*P*-value/OR (95% CI)/HR (95% CI)	Findings for mortality/mRS score
Bentes et al. 2017 (6)	IV-rtPA	101	1 year	6 (5.90%)	*P* = 0.213			At the 12-month, mRS was greater in patients treated with IV-rtPA than in patients not treated with IV-rtPA (57.4% vs 38.8%), *P* = 0.032
	Control	50		6 (12.00%)				

Belcastro et al. 2020 (7)	IV-rtPA and/or MT	262	7 days	10 (3.80%)	*P* = 0.450			
	Control	254		6 (2.30%)				

Alvarez et al. 2013 (8)	IVT	12	3 months	3 (25.00%)				An insignificant difference in poor outcome (mRS > 2) was observed among patients with and without seizures (*P* > 0.05) at three months

Kuohn et al. 2022 (20)	Overall	595,545	Median = 4.66 years			5,500 (2.20%)		
	IV-rtPA	22,818				283 (3.20%)	HR = 1.50 (1.39–1.63, *P* = 0.001)	
	MT	802				13 (4.11%)	HR = 1.86 (1.86–2.38, *P* = 0.001)	
	IV-tPA + MT	1,139				18 (4.27%)		
	Control	570,786				5,186 (2.16%)	Ref	

Xu J et al. 2023 (21)	Standard-dose IV-rtPA	966	Not mentioned	11 (1.10%)	*P* = 0.200			Higher in-hospital mortality was observed in low-dose as compared to standard-dose (1.1% vs. 0.5%), AOR = 1.93 (0.47–7.90), *P* = 0.36

Brigo et al. 2020 (22)	IVT	558	7 days	25 (4.48%)				
	IAT	122		7 (5.74%)				

Brigo et al. 2020 (23)	IVT	51	7 days	25 (31.6%)				mRS was significantly higher in patients with seizures as compared to patients without seizures (median: 4, IQR: 2–5 versus median: 3, IQR: 2–4) *P* = 0.007
	IAT	12		7 (8.90%)				
	MT	51		21 (26.60%)				

Tako et al. 2022 (24)	Overall	979	7 days	38 (3.90%)				Patients with early seizures had greater mRS score (3–5 points) at discharge as compared to patients with early seizures (87.0 vs. 49.3%) *P* = 0.017
	IV-rtPA	NA		17 (44.70%)				
	MT	NA		19 (50.00%)				
	IV-rtPA + MT	NA		12 (31.60%)				
	MT with TICI ≥ 2b	NA		17 (89.50%)				

Kohlhase et al. 2022 (25)	MT+/IV-rtPA	208	Not mentioned	6 (3.80%)	*P* > 0.050			Higher mortality rates were observed in the MT /IV-rtPA– group compared with the MT+/IV-rtPA+ and MT+/I-rtPA– groups, as well as a higher mortality rate in the MT /IV-rtPA+ group compared with the MT+/IV-rtPA– group
	MT+/IV-rtPA+	264		5 (3.20%)				
	MT /IV-rtPA+	169		6 (3.80%)				
	MT /IV-rtPA	346		9 (6.30%)				

Mushannen et al. 2021 (26)	IV-tPA	342	7 days	11 (3.20%)	*P* = 0.999			Early PSS-related mRS scores were higher on discharge, indicating poorer functional outcome, but the difference in mortality between seizure (9.1%) and non-seizure (4.3%) groups did not reach statistical significance, *P* = 0.134
	MT	70		2 (2.90%)				
	IV-tPA + MT	96		3 (3.10%)				
	Control	501		29 (5.80%)				

Ba et al. 2021 (27)	MT + IV-rtPA	1,638	7 days	60 (3.70%)	The analysis of 343 patients with and without rtpa showed no significant difference in early seizures occurrence, *P* = 0.386			About 6 (10%) patients with early seizures died, while 146 (9.3%) without early seizures died at 7 days, *P* = 0.846

Agarwal et al. 2021 (28)	Overall	291	3 months	37 (12.70%)				Patients with early seizure had significantly higher mRS score at 3 months follow-up compared to those without (3 vs. 2), *P* = 0.001
	MT	Not given		*n* = NA				

Agashe et al. 2021 (29)	MT	98	90 days	4 (4.10%)				Patients with early seizures are eight times more likely to have poor functional outcomes (mRS > 2) at 90 days compared to those without seizures

Zöllner et al. 2020 (30)	IVT	13,356	Not mentioned	199 (1.50%)	IVT+ versus IVT – patients, *P* = 0.070			
	IVT	13,356		237 (1.80%)				
	IVT + MT	1,013		17 (1.70%)	IVT+MT+ versus IVT +MT– *P* = 0.999			
	IVT + MT+	1,013		17 (1.70%)				

Anadani et al. 2019 (31)	MT	459	7 days	11 (2.40%)				The occurrence of seizures was associated with 90-day mortality and poor functional outcome (mRS > 2)

Eriksson et al. 2023 (32)	MT	2,120	3 months			120 (5.70%)	HR = 0.51 (0.41–0.65, *P* = 0.001)	
	IVT	1,535				134 (8.70%)	HR = 0.81 (0.65–1, *P* = 0.001)	
	Control	1,545				156 (10.10%)	Ref	

Nesselroth et al. 2018 (33)	IV-rtPA	141	2 years	7 (5.00%)	OR = 64% decrease in the risk of developing early seizures shown when treated with r-tPA, in comparison to other treatments, *P* = 0.001			

Keller et al. 2015 (34)	IV-rtPA	302	Median = 42 months			42 (13.9 %)		

Gruber et al. 2023 (35)	MT	348	Median = 77.6 months	14 (4.00%)		32 (9.00%)		

Alemany et al. 2021 (36)	MT	344	5 years	21 (6.10%)		14 (4.12%) in the 1st year, 10 (3.7%) in the 2nd year, and 4 (1.6%) in the 5th year. The 5-year cumulative incidence = 9%		After MT 53 (15.40%) died in the acute phase, and 13 (4.46%) died during the first year Early seizure and/or PSE does not increase acute or long-term mortality

Naylor et al. 2018 (38)	IAT + IV-rtPA	112	2 years			5 (4.5%)		Significant differences in mRS (0–2) were observed at 3 months among groups, i.e., IAT+IV-rtPA = 52%, IAT = 39%, IV-rtPA = 52.6% and control = 61%, *P* = 0.001
	IAT	93				12 (12.9%)		
	IV-tPA	363				21 (5.8%)		
	Control	1,375				27 (2%)		

Ferreira-Atuesta et al. 2021 (40)	IVT	1,225	Median = 1.6 years	*n* = NA (6.00%)	OR = 1.52 (1.09–2.08) *P* = 0.008	*n* = NA (8.00%)	HR = 1.15 (0.80–1.65, *P* = 0.43)	
	IAT			*n* = NA (7.00%)		*n* = NA (5.00%)		
	MT			*n* = NA (8.00%)	OR = 2.11 (1.28–3.31) *P* = 0.001	*n* = NA (5.00%)	HR = 1.4 (0.56–3.79), *P* = 0.42	
	Control	3,004		NA	Ref	NA	Ref	

Eriksson et al. 2020 (41)	MT	90	Median = 1,070 days	4 (4.40%)		4 (4.4%)	Risk of PSE was 3.9% (95%CI: 0.0–8.2%) at one year and 5.3% (95%CI: 0.2–10.4%) at two years	

Brondani et al. 2019 (42)	IV-rtPA	153	2 years	4 (2.60%)		17 (11.1%)		Among 21 patients, 14.3% of the patients died

Arntz et al. 2013 (43)	IV-rtPA	537	Mean = 9.8 years			3 (8%)		A poor outcome (mRS > 2) was significantly higher in patients with epilepsy as compared to patients without epilepsy (27.5% vs 9.8%), *P* = 0.001

Chen et al. 2018 (59)	IV-rtPA and/or MT	474	2 years	7 (1.70%)		32 (7.8%)		

Lei et al. 2021 (64)	MT	152	90 days	12 (7.90%)				Out of the 96 patients who survived treatment, 42.11% experienced poor functional outcomes (mRS 3–5)

Chen et al. 2017 (65)	IV-rtPA	348	Median = 559 days			22 (6.3%)		

ASS = acute symptomatic seizure; ASPECT = Alberta Stroke Programme Early Score; PSE = post-stroke epilepsy; IV = intravenous; rtPA = recombinant tissue plasminogen activator; MT = mechanical thrombectomy; IVT = intravenous thrombolysis; IAT = intra-arterial thrombolysis; mRS = modified Rankin Scale; RTs = reperfusion therapies; TICI = thrombolysis in PSS = post-stroke seizure; OR = odds ratio; HR = hazard ratio; NA = not applicable
